# Epidemic Cholera: Miscellaneous Observations—Historical, Statistical, Ætiological and Therapeutic, on the Prevailing Epidemic

**Published:** 1833

**Authors:** Daniel Drake

**Affiliations:** Cincinnati


### WESTERN JOURNAL

OF THE

### MEDICAL AND PHYSICAL SCIENCES.

JULY, AUGUST AND SEPTEMBER, 1833.

### Essays and Cases.

### Art. I.—Epidemic Cholera.

*Miscellaneous Observations—historical, statistical, ætiological and therapeutic, on the prevailing Epidemic, by* Daniel Drake, M. D.

In the short space of fifteen years, more books, pamphlets, tracts, discourses, reviews, proclamations, and advertisements, have been published on and about the Cholera, than on the subject of most other diseases, from the days of Hippocrates to the present hour. No malady ever before set so many pens in motion, or provoked so much repetition. The novelty and fatal character of the disease have given origin to the multiplicity; the uniformity of its symptoms, progress and termination, to the repetition. To which may be added the obscurity which hangs over its remote cause, and the difficulty of treating its confirmed and advanced stages, both of which have incited all classes of the profession to speculations on its ætiology and therapeutics. The Western Journal, among others, has been fraught with these matters; and the present number seems destined to go forth, with as many *verba cholerica* "lumbering at its back" as encumbered any of its predecessors.

### 1. *Spread of the Epidemic.*

In the last number it was announced, that the Cholera was spreading off from the Ohio in either direction. Its progress in the south has been rather indefinite. In Kentucky it seems not to have extended, with violence, beyond the central towns of Lexington, Danville, Harrodsburg and Bairdstown. It has, indeed been more decidedly severe in the northern, than the southern half of that state. The towns which have suffered most, are Maysville, Flemingsburg, Cynthiana, Georgetown, Paris, and Lexington; but most of the villages north of the Kentucky river have been afflicted with more or less severity, and the whole intervening country has had its visitation. As yet the facts necessary to a history of its geographical range in that state, and its comparative violence in different parts, have not been placed, in an authentic shape, before the profession.

I have still fewer materials for its history in Tennessee, where, however, it seems to have prevailed with less intensity than in Kentucky. But Shelbyville is an exception to this remark, as, from the newspaper accounts, its mortality in that town was very great.

Illinois and Indiana, it would appear have suffered but little. The eastern portions of the latter state, have been most afflicted. Some villages have been scourged, while the greater number have entirely escaped.

Ohio has suffered more than any of the states enumerated, except Kentucky. The shores of the river and the valleys of the Scioto, and the Little and Great Miamies have been most affected. It is certainly a remarkable fact, that the eastern half of the state has almost escaped. Lancaster, Granville, Newark, Zanesville, and the other towns east of the Scioto, have not been reached; while Chillicothe has had an average attack, and Columbus a most mortal and protracted visitation. Portsmouth, at the junction of the Scioto with the Ohio, forty miles below Chillicothe, and Circleville, half way between the latter town and Columbus, appear to have suffered little or none at all. Of the towns in the Miami country, Urbana, Springfield, Xenia and Hamilton, have either escaped, or been but slightly visited; while Lebanon, Dayton, and particularly Troy, have experienced a considerable mortality. Many small villages, as far as I now know, have entirely escaped. The visiters at the Yellow Springs, amounting generally to 150, continued throughout the whole watering season, entirely exempt. Of all the neighborhoods or towns of the Miami country, none has been so fatally affected, as the scattering village of Sharon, and its vicinity, twelve miles north of Cincinnati; where it appeared suddenly in the month of July, and in the course of a week, swept off an almost unprecedented number of its little population. An entire family consisting of eight persons died in quick succession. Three other and larger villages, Reading, Springdale and Montgomery, lying in different directions, within three or five miles, suffered but little.

Throughout the whole Valley of the Ohio, from the table land of our own state, and Indiana, to the banks of the Tennessee, the disease seems to have nearly subsided. It might be said to have risen and fallen, with the hot weather, though it appeared in some places before the *summer* heats, and still continues in others since they have ceased. Of its rise and fall in Cincinnati I shall say something presently.

### 2. *Ætiological Remarks.*

It seems to me impossible, not to feel a sentiment of wonder, at the unequal power with which the epidemic visits different places in the same region of country. How can this be explained? Why should two villages, under the same topographical, climatorial, and social circumstances, within a few miles of each other, be affected in such very different degrees? As Troy and Urbana, Dayton and Springfield, Lebanon and Xenia, Columbus and Zanesville; or Flemingsburg and Mayslick, only ten miles apart in Kentucky? And why should Lexington suffer so much more than Frankfort, the capital of that state, though only twenty-five miles distant from each other? Lastly, why should Cincinnati for two years be so much more fatally invaded, than Louisville, when the topographical and commercial circumstances of the two places are so nearly alike?

None of the prevailing hypotheses can explain these irregularities. Let us take that of intense heat,—were not all these places exposed equally to it? But the heat of the preceding summer was not uncommon.

The resort to copious rains in May and June, would avail quite as little, for in other years the same rains have occurred without producing Cholera; and during the present, they fell on all these places alike. Thus Xenia and Lebanon, are only twenty-four miles asunder, but the former has nearly escaped and the latter has been severely visited.

The miasmatic hypothesis is equally at fault; for in the first place, the same sources of miasmata have existed for years in the very region we have travelled over, without producing Cholera; and secondly, different places, bearing an exact resemblance, as to sources of miasmata, have been visited in very different degrees. Thus Dayton and Hamilton, bear the same relation to the great Miami and its valley; are nearly of the same age and size, and are kept in much the same condition; but Dayton has been far more fatally afflicted than Hamilton. And Columbus, Delaware, Circleville, Chillicothe and Portsmouth, all lie on the Scioto river; but the first has lost more of its inhabitants, than all the rest taken together. Still further, Covington and Newport lie adjoining, in the valley opposite this city, and the former has been much more affected than the latter. Further, a great number of towns and villages in Kentucky, are situate in topographical circumstances almost identical with Flemingsburg, Paris and Lexington, without having been scourged like them, and some have lost scarcely a single inhabitant. Maysville and Louisville bear the same relation to the river, but their sufferings have been widely unequal. Aurora and Rising Sun, in the state of Indiana, are both on the bank of the Ohio—the former has nearly or quite escaped, the latter has had a visitation. Cynthiana and Bairdstown are much alike in topographical circumstances, but suffered very unequally. Lastly, some towns both in Ohio and Kentucky, which are so situated, that copious rains might be said to be necessary to promote putrefaction, suffered but little, while others in which such rains would bury the decomposable matter, in and about them, suffered much. Thus, in every aspect under which the miasmatic hypothesis can be viewed, in reference to the Western Country, it appears to me untenable. But the difficulties which environ it, connect themselves equally with other countries; for what should cause malarious situations to send forth, *progressively*, from the Ganges to the Mississippi, within the last few years, the miasmata which are said to produce Cholera? This single fact must overturn the hypothesis.

The theory of contagion is equally unsustained by the facts recently developed. Cholerine has prevailed extensively in the West, affecting, in many places, more than half the population, and coming upon them simultaneously. How can this be referred to contagion? How often have we seen a single member of a family taken off, and all about him preserved from a similar attack! If contagious, how could so many of the exposed escape? What rubbing of the surface of the sick, and impregnation of the clothes and bedding, with their perspiration—what repeated examination of their alvine excretions—what concentration of friends about their death bed, without, in many cases, a single one of the whole thus exposed to their exhalations, experiencing an attack! If contagious, why have neighborhoods and villages, which had perpetual communication with places where it reigned, remained uninfected? Finally, why has the disease already nearly ceased, although its subjects are indefinitely unexhausted?

The electrical hypothesis is equally unsupported. Those who maintain it, are bound to make out their case; but this has not been done. Most of the summer has been no way remarkable for electrical phenomena in the atmosphere, nor for their absence. Indeed, nothing indicates to us that the electricity of the earth and air has been disturbed in its equilibrium beyond the degree of former irregularities.

The theory of its aqueous origin, is equally unsatisfactory. In this city, the inhabitants drink river water, in Lexington and Georgetown, well and spring water, issuing in veins from between limestone rocks, yet the cholera prevailed in both; while many villages and country settlements, have remained comparatively exempt; although supplied with the same kind of water with others in their neighborhood, which were severely afflicted.

It has been said by some writer of Europe, that the disease has only prevailed in alluvial, diluvial and secondary formations, and is therefore in its cause, connected with these geological tracts. But what others are inhabited? I know of none. Undecomposed slate or granitic rocks, are uninhabitable. Man must necessarily reside on the secondary and alluvial. New York, Philadelphia, and Baltimore, lie in primitive or granitic regions; but their immediate sites are alluvial and diluvial beds of loam, gravel, sand and pebbles, chiefly the *debris* of primitive and transition rocks. If these deposites had not been made, those cities would not have been built, and so of all others throughout the world. Hence the cholera is confined to the formations and deposites, which are newer than the primitive, merely because man is found no where else. The cause may exist over other formations, but the effect on man cannot, because man is not there. West Point, embosomed in a granitic mountain, has, it is true, remained exempt; but many other places in secondary regions, as Zanesville, in this state, have not been visited; and West Point is an alluvial bed, of precisely the same composition, with that of New York. Finally, those who maintain the hypothesis of exhalation from secondary and tertiary formation, are bound to assign some cause for their *gradual* development from India to Louisiana, which they have not done.

The supporters of a progressive meteoration, or vitiated condition of the atmosphere, from intestine changes in its occult principles, should feel it incumbent on them, to go beyond the mere invention of a name. Till they do this, they have done nothing more, than express the fact that the disease spreads, from the agency of a cause which is connected with the atmosphere, as one of its modes of existence. It is certainly not possible to disprove the existence of this condition, but sound philosophy does not call for such disproof. I think it will be admitted, however, that if this meteoration be a state of the atmosphere itself, the disease ought to spread according to the direction of the winds; and that when two villages are contiguous, both should be affected at the same time or in rapid succession, instead of which, it is not uncommon, as we have witnessed, lately, for one entirely to escape, while the other is mortally scourged.

It seems scarcely necessary to say, that no reference to the sensible qualities of the air, can account for the origin of the Epidemic. It has prevailed under the most opposite conditions. What resemblance is there between the climates of Calcutta and Moscow—of London and New Orleans? Again, the first occurrence of the disease in this city was in October—the second in summer. Further, the state of the weather for the last three or four months, has been the same at the same time, over the different parts of Kentucky and Ohio, while some spots have been invaded by the Epidemic, and others have gone free. The whole season has been uncommonly productive in all the fruits, grains and culinary vegetables, which grow, spontaneously, or are cultivated, in this region, and they have ripened in great perfection. Lastly, the season has not been unprecedented in any of its characteristics.

On the whole, it appears clear to my apprehension, that all our knowledge of the cause of Epidemic Cholera is purely negative. We know what it is not, but have not yet learned what it is. In this state of ignorance, it may seem presumptuous to speculate on the question, whether the Cholera will become one of the permanent diseases of our country. Many people fear it will. I do not myself cherish this apprehension. The world has been repeatedly visited by wide-spreading and mortal pestilences, and they have generally continued for several years. The Cold Plague, or Spotted Fever, (Pneumonia Typhoides,) began in the valley of the Connecticut river, in the year 1806 or 7, and reached that of the Ohio in 1814. It continued in some part of the United States for 8 or 10 years. For the last 15 or 18 years, it has not been met with. In Russia, where the Cholera began in 1830, it seems already to have disappeared; as will no doubt be the case, in the United States, in the course of another year.

### 3. *Exciting Causes.*

It remains, I think, to be ascertained, whether the dietetic edicts of the profession, have done much to prevent the Cholera. I am persuaded, that a reduced and inadequate diet, has sometimes invited the disease; and have not as yet met with many cases, in which it was excited by the use of the prohibited vegetables. Certain it is, that the malady has repeatedly invaded those who carried themselves according to the strict letter of the law, and abhorred all *constructive* interpretations. In framing rules of diet, for the respective communities in which they practise, physicians have copied from each other, and most persons have thought it best to err on the "safe side." That many individuals have suffered in their health, from change of diet, and the omission of all succulent and acid fruits, is, I think, quite certain; though it cannot be asserted, that they were not thus preserved from the Epidemic.

Changes of weather from dryness to moisture, and from heat to cold, have undoubtedly been exciting causes. Thus in the decline of the late epidemic, in this city, a sudden change of weather more than doubled the number of deaths in 24 hours, and produced much inquietude in the public mind; but the more discerning thought they saw nothing in it, but the influence of a new exciting cause; and the rapid abatement of the disease immediately afterwards, till it entirely ceased, proved that the epidemic influence was indeed declining at the time.

Of all known exciting causes, the greatest is fear. The following fact was communicated to me by a gentleman of veracity. An inhabitant of the state of Mississippi, was taken on board a steamer descending to New-Orleans. In a few minutes afterwards, he learned that some of the passengers had the Cholera. Becoming at once greatly terrified, he begged to be set on shore in the woods, but his request was not granted. In four hours he was seized with the disease, and died the same day.

#### 4. *Treatment of the Epidemic in Lexington.*

The following abstract was prepared for the Analytical head, but I prefer to introduce it here.

Professor Yandell, in the 22d No. of the Transylvania Journal, has favored the profession with a history of the late Epidemic in Lexington. We shall give our readers a brief abstract of his treatment.

*First stage.* Rest, warmth to the skin, calomel in doses of 20, 60, and 120 grains. Generally successful. In some cases a *speedy* emetic.

*Second stage.* Calomel in larger doses. An ounce divided into three portions, in desperate cases. In a few cases an emetic. This course was generally successful. But in addition, epigastric stimulation, laudanum, ice and iced lemonade, were used with advantage. The vegetable astringents sometimes did good.

*Third stage.* In some cases calomel in large doses. Ice. Diffusible stimulants, but generally with disappointment in their effects. The cold dash did not succeed. This stage generally proved fatal.

He seldom employed blood-letting in any stage of the disease; thinks that it has done harm, but is sometimes beneficial.

In the succeeding number of the Journal just quoted, Professor Cooke has stated his method of treatment. It may be said to resolve itself into the administration of calomel. Dr. C. has observed, as be thinks, that, for several years past, there has been an increasing necessity for larger doses of calomel in the treatment of our summer and autumnal endemics. Regarding cholera as one of these, and the most violent among them, he was led to administer that medicine, in what we suppose the profession generally, will regard as *maximum* doses. Sixty grains he found too small, even in mild cases, and preferred one hundred and twenty; while violent attacks called for an ounce, to be repeated until the patient should take a thousand or fifteen hundred grains. But even these doses were found insufficient, in some instances, and table spoonful (ounce and a half) doses were repeated several times. In one case, which terminated favorably, the patient took a table spoonful every six hours. The first day four were taken, the next three, and three others were also administered to supply the place of three that were supposed to be thrown up. If but one-third of each of these was retained, the patient kept down eight, or an apothecary's pound, the largest quantity, we presume, hitherto administered in the practice of medicine. It was partly in reference to such doses as these, that we ventured in our last number, to express the opinion that some portion of the extraordinary mortality of the citizens of Lexington, (500 out of 6000) might be owing. It appears, however, from Dr. Cooke's paper, that from absence and sickness, he did not treat a great number of patients, and that his brethren did not administer calomel with equal boldness. We have seen, indeed, and set forth in our last number, that Prof. Dudley gave that medicine in 10 and 20 grain doses, and placed his chief reliance on emetics. These, however, Dr. C. seldom employed, and seems to have thought every thing except calomel, unworthy of confidence. We must not conclude this notice of Dr. Cooke's paper, without adding that the method he pursued was generally successful. We are not at liberty nor disposed to question this statement, but may ask whether those who recovered would not have got well, if but a tenth part of what they took had been administered?

In addition to these publications of Drs. Cooke and Yandell, and of Dr. Dudley, noticed in our last number, we have received from Dr. T. B. Pinkard, of Lexington, an account of his observations on the treatment of the Epidemic. The Doctor states his experience to have been extensive.

Among his means of prevention was a little good old brandy or whiskey, after fatigue, or when mild incipient symptoms existed, and he speaks highly of its benefits. "As a medicine," says he, "I prescribed it to arouse the dormant energies of the nervous system, assist digestion, invigorate the languid circulation, and remove feelings of mental and corporeal exhaustion. It is a remarkable fact, that during the epidemic, many individuals of both sexes, who had never been accustomed to any alcoholic stimulus, and to whom even the taste of brandy or whiskey was disagreeable, found its effects to be those of a valuable cordial and tonic, without producing any unpleasant sensations in the brain, or flushing of the face; and it was also observed by those who thus used it as a medicine, that as the epidemic declined, its effects became disagreeable, so that it was most willingly discontinued. That it was, in fact, useful to many under proper circumstances and in regulated doses, I am well assured; but it is right that I should add, that the intelligence having gone abroad, that our physicians had recommended brandy as a preventive of Cholera, many persons considered it as a license to indulge their depraved appetites for stimulation, and by excessive drinking, not only invited the disease, but rendered it much more violent and mortal than it would otherwise have been."

We do not consider the profession liable for these abuses. It is their duty to give *correct* advice, and they should not be held responsible for such consequences as Dr. P. has revealed to us. Under this impression I shall state, that the visitors at the Yellow Spring, male and female, drank ardent spirit as a preventive, daily, beginning in the morning before breakfast, and all of them escaped the Epidemic, while it was prevailing, as we have seen, in many parts of the Miami country.

For the cure of the disease in its first or diarrhœal stage, Dr. P. gave, every four hours, 15 or 20 grains of calomel, with 5 of ginger or cinnamon, 1 or 2 of capsicum, and 5 of camphor, in a powder, with loaf sugar, washed down with brandy and water, or pepper tea; and confining his patients to a warm bed, allowed them ice, or iced water, with camphorated spirit. If the stomach was loaded, he administered an emetic of ipecac or mustard, and worked it off with tepid salt water; after which he allowed them a moderate dose of laudanum or opium, and gave the powder just mentioned. When the pulse was strong and the patient of a full habit, he bled till the morbid energy of the circulation was reduced. When the diarrhœa was subdued, he permitted a bitter infusion, and a regulated diet.

In the second or choleric stage, attended with spasms and rice-water discharges, he often found an emetic of service; his dose of calomel was 20 or 30 grains, with one of opium every hour; he gave iced soda water; applied external irritants, and administered injections of starch and laudanum. He also wiped and rubbed the skin: But his chief reliance was on calomel, given in the above doses, which he regarded as preferable to larger ones, till the functions of the liver were restored. However, he found when the serous discharges were profuse, that opium was of great service.

In the third stage, his greatest dependence was on ice and iced water, which he allowed *ad libitum*· he also gave calomel, but found the ice far more beneficial; indeed, it was his principal remedy.

### 5. *Cholera in Cincinnati.*

The July number of this journal contains so much on the subject of the visitation of Cholera, under which the city was then suffering, that I shall say but little of it at this time. The Epidemic attained its maximum in the last week of the month, on the 24th of which there were thirteen interments from that disease. By the first of August it began to abate and by the beginning of the present month, September, had nearly ceased. But I shall be a little more specific, and present the following tabular view, from the returns of the sextons to the City Council, which, however, I fear are not very accurate.

Deaths in Six Months.

| 1833. | Whole No. | Cholera. | Scarlatina. | Dysentery |
|---|---|---|---|---|
| April, | 50 | 2 | 11 | 0 |
| May, | 107 | 38 | 3 | 3 |
| June, | 154 | 78 | 8 | 2 |
| July, | 373 | 187 | 9 | 3 |
| August, | 177 | 61 | 11 | 2 |
| September, | 95 | 4 | 2 | 3 |

From this table it appears, that the total number of deaths from the late visitation of Cholera, was 370, from Scarlatina 44, and from Dysentery 13.

If we add the loss from the Epidemic to that of last year 571, we have an aggregate of 941, or about three out of every hundred of our entire population. This loss, although great in absolute degree, is much less in proportion to our population, than that of many smaller towns in the West, as for example, Columbus, in this state, and Flemingsburgh and Lexington in Kentucky. I have not seen a statement of the entire loss of Columbus, but it is admitted to have been very great. That of Flemingsburgh was 66. If its population was 1000 (too high an estimate perhaps) the loss was more than six out of every hundred. That of Lexington was 500, or about eight of every hundred, taking the population of the town at 6000. The relative loss of Flemingsburgh and Cincinnati, was as two to one, of Lexington nearly three to one, counting two visitations to us, and but one to each of those places. Much of this difference was no doubt owing to a greater popular panic (which I believe to have existed) there than here, but it cannot be said that the remote cause was not more virulent.

The whole number of deaths in Cincinnati, for six months beginning with April, in the years 1831, '32, '33 will appear from the following table:

| | 1831. | 1832. | 1833. |
|---|---|---|---|
| April, | 55 | 77 | 50 |
| May, | 45 | 110 | 107 |
| Jnne, | 92 | 95 | 154 |
| July, | 102 | 116 | 373 |
| August, | 128 | 141 | 177 |
| September, | 93 | 97 | 95 |
| Total, | 515 | 636 | 956 |

If we deduct from the mortality of the present year the Cholera returns, we have a remainder of 586, which is little more than the mean term of the two preceding years.

Among the complications with Cholera this summer, the most important was dysentery. The number of cases has been considerable, though the deaths for six months were only 13. In some instances the disease commenced as Cholera, and terminated in dysentery—in others the reverse; while a few exhibited many of the characteristic symptoms of both, at the same time. In proportion as the dysenteric character predominated, was the safety of the patient. The treatment which this dysentery required, was substantially the same with that of Cholera. Calomel and opium combined, were the indispensable parts of the *methodus medendi;* but mucilages—oleo-saccharums—cretaceous mixtures, and the different preparations of rhubarb, were valuable auxiliaries. In some cases, astringents were of service.

Having in our last article on Cholera, gone fully into the treatment then in vogue here, I need only add, that it continued much the same, till the disappearance of the disease.

In conclusion, I shall repeat for the hundredth time, that all who would be cured of Epidemic Cholera, should commence the treatment in its first or diarrhœal stage. Almost any method, provided rest be a part, will then succeed; while no means yet discovered, can be relied on, after vomiting and spasms have supervened; and this is all that is of any substantial practical value, in the thousand and odd volumes which have been written on this malady. Such at least is my opinion.

### 6. *Fatal Cases.*

The publication of fatal cases of Cholera, may, perhaps, throw some light on the causes of its mortality. The following are all that have happened in my practice, since the reappearance of the Epidemic in April last. They are stated in the order of their occurrence.

Case I. Mrs. H. aged 64. This lady had been for several years afflicted with winter bronchitis, which had at length become habitual, and was occasionally accompanied with diarrhœa. Both in mind and body she exhibited strong marks of senescence for one of her years.

On the afternoon of the 14th of May, 1833, the atmosphere being cool, with N. E. wind, and more or less rain, she was attacked with diarrhœa serosa. It continued through the night.

Next morning, 15th, at an early hour, I was called in. The diarrhœa continued, with nausea and occasional vomiting. The alvine discharges were gruel-like: the gastric, watery and mucous: no bile in either. Her pulse was weak and slow and empty; hands shrivelled, complexion dark, lips purple, and eyes sunken. She had much thirst and considerable cramp. These symptoms continuing and increasing, she expired in the evening.

As this lady had been subject to diarrhœa, a choleric tendency was not suspected by her friends, and but little was done through the night. In the morning she was passing into a state of collapse. Opium and calomel, with gentle internal stimulants and stronger external, were tried, but had no effect in arresting her malady.

At the time of her attack there was but little cholera in the city. Her bronchitis afforded no protection from the choleric poison, although it was attended with hectic fever. The state of the gastro-enteric mucous membrane, which every now and then gave her diarrhœa, seems to have favored the production of the cholera. The change in her complexion was early, compared with the quantity of alvine evacuation, which arose, perhaps, from the disorganized state of the pulmonary membrane, a condition unfavorable to the due aeration of the blood.

How far a case of this kind is *necessarily* fatal, I cannot pretend to say; but is there not much reason to believe, that, if it had been treated with the usual remedies in the first stage of the attack, it might have terminated favorably? I am disposed to think it might.

As this lady was confined to her chamber, in a high and healthy part of the city, had seen no one with cholera, and was exposed to the action of no local miasm, her case presents a distinct and palpable fact against the doctrines of contagion, and malaria.

Case II. June 8th. The infant child of W. R. M. aged one year. This patient was supported by a dry nurse, and exhibited considerable emaciation. It was subject to occasional diarrhœa and vomiting. On the morning of the 8th, it was attacked in this way, and took some small doses of calomel, by the order of another physician, in my absence. In the evening when I saw it first, although its evacuations had not been profuse, its pulse was very small and its feet and hands cold and purple. No external heat could warm them. It perpetually rolled its head, like a patient with hydrocephalus. Its pulse continued to sink; in the night it had spasms, and before morning expired. Calomel and opium were repeatedly administered, and external stimulants applied but without effect.

In this case the epidemic was invited by the previously disordered state of the bowels. There was much in its physiognomy and symptoms, not very expressible, to distinguish it from the closing stages of cholera infantum. After the diarrhœa began, several hours elapsed before any medicine was exhibited; but perhaps the case would have proved fatal under the earliest treatment.

Case III. Mrs. H. aged 45.—Of a nervous temperament. This lady was subject to high degrees of hysterical irritation, attended with great cerebral excitement. She labored habitually under great apprehensions of the epidemic. Being on a visit to the country, she became indisposed with head-ache and feverish commotions. In a state of anxiety and alarm, she returned to the city. By the aid of cathartic medicines, containing calomel, cold applications to her head, and subsequently, the sulphate of morphia with sulphuric ether, she soon got much better and went about her chamber.

On the morning of the 20th of June, being rather costive, she took a strong infusion of senna, which began to operate about ten o'clock. Between twelve and one o'clock she went down to the dining room, and ate a hearty dinner, of soup, smoked beef and other articles. Soon afterwards she vomited violently, the discharges from the bowels became more profuse, and assumed the rice-water appearance. I did not see her till five o'clock.

Her pulse having considerable activity, I determined to bleed her. Keeping her in the bed, her feet were placed and kept for three hours, in hot salt water; a large vein in the ancle was opened, and blood almost as florid as that of the arteries, flowed in a full stream, which was not stopped until her pulse began to faulter, and syncope was threatened. Her circulation did not afterwards acquire the energy, which it possessed before the bleeding. As soon as I saw her, I gave her between 20 and 30 grains of calomel, which dose was repeatedly administered afterwards, with more or less laudanum—not, however, in such large quantities, as it is frequently given. Weak lye of wood ashes was likewise exhibited, and soda water allowed for a common drink. Pellets of ice were, however, more agreeable to the patient, and were allowed *ad libitum.* From the beginning, lumps of ice, in linen wrappers, were kept on the epigastric and umbilical regions, for more than twenty-four hours. The patient liked them much, though she constantly answered that they did not feel cold, except when they were moved to a new spot, and the sensation was then agreeable to her.

Under this treatment the vomiting nearly ceased, and her stomach at length bore strong coffee, for which she expressed a desire, and which was allowed her in small quantities. The diarrhœa continuing profuse, injections of coffee, starch and laudanum were administered, with the effect of restraining it. Twenty-six hours after the ice was applied to the abdomen, it was removed, immediately after which, the stomach became more irritable. Her pulse in the course of the attack sunk very much, and her hands, on the second day became very cold; but her feet retained their heat till near the close of life, which was not till the 25th, five days after the commencement of the disease.

In the course of this time, when the vomiting and diarrhœa had become much mitigated, a considerable tinge of bile appeared in the excretions, and hopes were entertained that she would recover. It was soon perceptible, however, that her eyes were suffused, her visage red, and her mind wandering. In short, the stage of consecutive fever, attended with determination to the brain was developed. Ice was now applied to the scalp, and stimulants administered more sparingly; but the redness of her face gradually changed to a darker hue, the delirium increased, a considerable degreee of coma supervened, her pulse gradually sunk, and the coldness of her extremities increased, till she expired. She scarcely had any cramp throughout the whole course of the disease.

*Remarks.*—This patient like the two preceding had not been within the atmosphere of any one laboring under that disease. Her temperament favored the action of the atmospheric poison, and explains the production of the cerebral affection, which fifinally caused her death. The immediate exciting causes of her malady, were, however, the cathartic, and the miscellaneous and excessive dinner which she ate during its operation. Had she after the *first* evacuation, laid down, and taken a liberal dose of laudanum, the cholera would not in all probability have been developed. In this case the refrigerant treatment had a pretty full and fair trial. It probably prolonged her life, by deferring her death from the stage of collapse to that of consecutive fever. Had she not been subject to paroxysms of cerebral irritation attended with great and sudden influxes of blood into the brain, she probably would have escaped the affection of that organ, and having been preserved through the previous stages, by the blood-letting, ice, calomel, and saline drinks, might have recovered. This was the first case in which I employed ice externally with perseverance and to much extent. As far I was able to estimate its effects, they were salutary, in relieving the irritability of the stomach and mitigating the epigastric distress. Such was the conviction of the patient. Before it was applied, the vomiting was incessant—after its discontinuance the nausea and retching became much worse than while it was used.

Case IV. On the 25th of June, Mrs. K. aged about 21, the daughter of the last patient, fell a victim to the same disease. She resided in the country and had been ill, with what form of disease I do not know; and was infirm and feeble when she was summoned to the city, on account of her mother's sickness.

From the time of her arrival, she was weak, anxious, depressed in spirits, and more or less inclined to diarrhœa; for which she took the blue pill, with weak lye and paregoric, with considerable relief. On the morning of the day on which she died, she was dressed and sat up. Early in the afternoon, her diarrhœa became profuse and serous. A large dose of ipecacuanha produced full vomiting, but brought away no bile. Ice was applied to the stomach, and calomel administered in doses of thirty or forty grains with opium. But nothing arrested the progress of her disease. She soon became affected with cramps, but not to a very great degree; her anxiety and restlessness were great, and her voice speedily sunk to a whisper. So early and great a degree of aphonia, I do not recollect to have witnessed in any other case. It attracted her own attention, and she inquired, in a voice scarcely audible, why it was that she could not speak louder. At length the characteristic complexion and coldness of the extremities came on, and she suddenly expired, a short time before her mother. The amount of alvine excretion was not very great.

*Remarks.*—In this patient the disease appeared to be excited by grief and apprehension. The remedies employed were useless. Could any of the others known to the profession have been more efficacious? The emetic did no good; the blood-letting none. The ice and calomel, and the alkaline solution, which was given freely with laudanum, were equally inert. Called to another case with precisely the same symptoms, I should perhaps follow the same practice, and most likely with the same result.

Case V. Mr. K. the husband of the last patient, a robust and healthy man, began to vomit immediately after leaving the death-bed of his wife, over whom he had exerted himself for six or eight hours. He had not previously complained to me, and, at the time, I regarded the disease as being suddenly excited by mental emotion. This was no doubt, in part, the case, but I have since learned, that two days before, he purchased and ate in the market, a dozen unripe pears; and that he labored under a diarrhœa, throughout the day on which she was sick, for which he took nothing.

His pulse being full and strong, he was bled nearly to fainting. The quantity taken was between sixteen and twenty ounces. An emetic of ipecacuanha was then administered which operated freely, but discharged no bile. Immediately afterwards, calomel and laudanum were exhibited freely, and he was allowed pellets of ice.

But the vomiting and diarrhœa continued. On the succeeding day his pulse being still active, he was, after I had had a consultation, bled a second time, and astringent injections with laudanum were administered. An alkaline solution was taken freely throughout the whole course of the disease. After the second bleeding his pulse became much weaker. The ethereal tincture of phosphorus, tincture of supatorium perfoliatum and laudanum, were then administered, and seemed to arrest his vomiting, but collapse came on and he died in forty-eight hours from the time when he became the subject of medical treatment.

*Remarks.*—In this case, the constitution of the patient and the symptoms of the disease seeming to invite it, I employed blood-letting to a greater extent, than in most other cases I have treated. The blood was quite as florid as in other diseases, but not sizy. The second bleeding was perhaps uncalled for, or even prejudicial. It demonstrated this, however, that copious venesection cannot cure cholera in all cases. To those who find in this disease, nothing but gastro-enteric inflammation, the issue of this case may be a useful lesson. Such an inflammation could scarcely survive such depletion, at an early period of the disease.

Case VI. On the 6th of July, I was requested about seven o'clock in the evening to visit Mrs. P. aged 60. At nine in the morning, having eaten nothing unusual at breakfast, she felt some pain in her bowels. About 11, a diarrhœa came on, and when I first saw her she said she must have had at least fifty motions. The discharges resembled rice water. Her pulse was still regular, and harder I thought, than could be explained by a reference to her age. Her stomach was quiet and she had no cramps. She had taken one dose of calomel and opium just before I saw her.* I immediately directed thirty grains of the former, and ordered her bled. My assistant, Mr. Wood, took about eight ounces, when she fainted. Vomiting came on, and she threw up the calomel. Another dose of the same weight was administered and retained; and the exhibition of large doses of that medicine with laudanum and weak lye, and the application of external stimulants constituted the chief treatment afterwards; with the addition, however, of astringent injections, which, more than any thing else, seemed to restrain the alvine discharges. She vomited occasionally. Cramps came on but were not very violent. Her pulse never recovered its energy from the time she was bled; and I am disposed to think the operation did her harm. It appeared to break up the tranquility of her stomach, which remaining, might have favored the action of calomel on the liver and sudorifics on the skin, under the action of which, it seems to me, she might have recovered. I have since, in a case remarkably analogous, refrained from bleeding, and the patient recovered under the use of forty grain bolusses of calomel, and large doses of laudanum, with diaphoretics. In the latter case, however, the medicines were administered only three hours after the diarrhœa commenced, while in the former, eight had elapsed, through the whole of which the discharges were profuse, and the patient allowed to rise from bed. Had she been bled six hours sooner, the operation would probably have been beneficial, or at least harmless.

*Conclusion.*

With this paper the Editor hopes he has concluded all that he need say on the Epidemic Cholera, except to review the publications of others. If he has appropriated a large portion of several numbers of the Journal to this subject, it should be recollected, that one of the chief objects of such a work, is to disseminate intelligence concerning new and fatal forms of disease. Among these the Cholera has been preeminent, and he has presumed that no other topic had such deep intrinsic interest.

Cincinnati, Sept. 30, 1833.

